# Tyrosine phosphorylation of Kv1.5 is upregulated in intrauterine growth retardation rats with exaggerated pulmonary hypertension

**DOI:** 10.1590/1414-431X20176237

**Published:** 2017-09-12

**Authors:** L.C. Fu, Y. Lv, Y. Zhong, Q. He, X. Liu, L.Z. Du

**Affiliations:** Department of Neonatology, the Children's Hospital, Zhejiang University School of Medicine, Hangzhou, Zhejiang Province, China

**Keywords:** IUGR, Kv1.5, PASMCs, Pulmonary hypertension, Tyrosine phosphorylation

## Abstract

Intrauterine growth retardation (IUGR) is associated with the development of adult-onset diseases, including pulmonary hypertension. However, the underlying mechanism of the early nutritional insult that results in pulmonary vascular dysfunction later in life is not fully understood. Here, we investigated the role of tyrosine phosphorylation of voltage-gated potassium channel 1.5 (Kv1.5) in this prenatal event that results in exaggerated adult vascular dysfunction. A rat model of chronic hypoxia (2 weeks of hypoxia at 12 weeks old) following IUGR was used to investigate the physiological and structural effect of intrauterine malnutrition on the pulmonary artery by evaluating pulmonary artery systolic pressure and vascular diameter in male rats. Kv1.5 expression and tyrosine phosphorylation in pulmonary artery smooth muscle cells (PASMCs) were determined. We found that IUGR increased mean pulmonary artery pressure and resulted in thicker pulmonary artery smooth muscle layer in 14-week-old rats after 2 weeks of hypoxia, while no difference was observed in normoxia groups. In the PASMCs of IUGR-hypoxia rats, Kv1.5 mRNA and protein expression decreased while that of tyrosine-phosphorylated Kv1.5 significantly increased. These results demonstrate that IUGR leads to exaggerated chronic hypoxia pulmonary arterial hypertension (CH-PAH) in association with decreased Kv1.5 expression in PASMCs. This phenomenon may be mediated by increased tyrosine phosphorylation of Kv1.5 in PASMCs and it provides new insight into the prevention and treatment of IUGR-related CH-PAH.

## Introduction

Pulmonary arterial hypertension (PAH) is associated with intrauterine growth retardation (IUGR), the fetal origin of adult disease (1,2). IUGR is defined as having a birth weight below the 10th percentile of the corresponding gestational age (3). IUGR predicts the risk of developing a variety of adult diseases including obesity, type 2 diabetes, and hypertension, as well as PAH (4,5). IUGR may also have a persistent effect on infant pulmonary vasculature, which when activated in adult life, predisposes one to a pathological response (6). Several clinical studies indicated that PAH is relatively common in extremely low birth weight infants (7,8). Rexhaj et al. (1) found that pulmonary hypertension induced by hypoxia in adulthood is associated with maternal undernutrition during pregnancy. In our previous study, we found that IUGR as a result of undernutrition is related to chronic hypoxia pulmonary arterial hypertension (CH-PAH) in adulthood, while the smooth muscle layer undergoes remodeling (2,9).

The pathological response of PAH primarily depends on the diameter of the resistance pulmonary artery (PA), such as thickening of the vessel wall and excessive constriction due to vasoactive substances (10). Control of the resistance PA rings depends on regulatory mechanisms intrinsic to pulmonary artery smooth muscle cells (PASMCs), such as the level of membrane potential. O_2_-sensitive voltage-gated potassium channels (Kv channels) including Kv1.2, Kv1.5, Kv2.1/9.3, and Kv3.1b in resistance PASMCs play a critical role in the maintenance of ionic homeostasis to modulate membrane potential under hypoxia conditions during PAH development (4,11–17). Recently, a number of studies demonstrated that Kv channels could be directly regulated by oxidative stress *in vivo* and *in vitro*, including through attenuation of Kv channel current and inhibition of expression of Kv channels (11). For example, gene expression of Kv channels, particularly Kv1.5, is decreased in a CH-PAH cell model (18).

Interestingly, the plasticity of Kv current in cardiomyocytes is associated with embryonic development (19). Studies indicate that ion channel activity is dependent on the phosphorylation status of the channel, and that tyrosine phosphorylation, in lieu of serine or threonine phosphorylation, is essential in regulating channel activity (20,21). Similarly, Huang et al. (22) found that with a selective tyrosine kinase agonist, the current density of Kv channels (*I*_K_) decreases in response to increased Kv1.2 tyrosine phosphorylation. Conversely, decreased tyrosine phosphorylation in Kv1.2 dramatically increases *I*_K_ (23). However, little is known regarding the effect of IUGR on regulating the phosphorylation status of Kv channels. Thus, we hypothesized that the tyrosine phosphorylation of Kv channels in PASMCs may be impacted by maternal nutrition during pregnancy.

In this study, we examined the mRNA and protein expression of the Kv channel α-subunit in an IUGR rat model, which was established by maternal food restriction during pregnancy with or without hypoxia exposure of the offspring in adulthood.

## Material and Methods

### IUGR and hypoxic pulmonary hypertension rat models

The IUGR rat model was established as described in our previous study (2). Female Sprague-Dawley rats weighing 250–300 g were selected to mate with male rats overnight. The females were then housed individually and randomly divided into two groups. Pregnant rats were fed standard chow either *ad libitum* or 50% of the *ad libitum* amount (as determined in our previous study) during the entire pregnancy. All rats were allowed to drink water freely. The offspring of the non-restricted mothers were considered the control group, while the offspring of food-restricted mothers (birth weight below 5.8 g, as previously determined) were considered the IUGR group. In order to avoid variability related to hormonal cycles in female rats, only male offspring were included.

The 12-week-old offspring were further divided into four groups: 1) control, 2) control-hypoxia, 3) IUGR, and 4) IUGR-hypoxia. The control-hypoxia and IUGR-hypoxia groups were housed in a hypoxia chamber (Biospherix Ltd., USA) for 2 weeks in which the fraction of oxygen was maintained at 10% by an oxygen controller (ProOx P360, Biospherix Ltd.), and with a constant temperature of 22±3°C. The control and IUGR groups were maintained under normoxia conditions during the same period.

All procedures and measurements were approved by The Animal Care and Use Committee of Zhejiang University, China.

### Mean pulmonary arterial pressure (mPAP) measurement

In order to measure the mPAP, rats were anesthetized with pentobarbital sodium (50 mg/kg body weight, intraperitoneally) and placed on a thermo-regulated surgical table. A PE-50 catheter with the angle directed anteriorly was inserted from the right jugular vein through the right heart into the main pulmonary artery. Placement at each stage was confirmed by respective pressure contours. Hemodynamic values were measured and automatically calculated by a physiological data acquisition system (Acknowledge MP150; Biopac System Inc., USA), as described previously (9).

### Immunohistochemistry and morphometric analysis

Left lungs were isolated from rats, inflated with ice-cold 10% formalin in phosphate-buffered saline, and fixed for at least 48 h at 4°C. Fixed lung tissue was paraffin-embedded, sectioned at 4–5 μm, and processed for immunohistochemistry. Samples were incubated overnight with a primary antibody against α-smooth muscle actin (α-SMA, Abcam, USA), followed by incubation with a secondary antibody (horseradish peroxidase [HRP] polymer) for 30 min at 37°C. At least five fields of view from different lung sections per animal were evaluated. Sections of peripheral lung were examined, and fields with large airways or major vessels were avoided. The percentage of smooth muscle layer present in the whole vessel was measured microscopically by Image Pro Plus software 5.0 (Media Cybernetics, USA).

### Quantitative real-time polymerase chain reaction (RT-PCR)

Total RNA was purified from smooth muscle tissue kept at –80°C according to the isolation kit protocol (Omega, USA). RNA was reverse transcribed using a reverse transcriptase kit (Takara, China). Quantitative RT-PCR was performed with a LightCycler 480 Instrument (Roche, USA) following the Takara SYBR-Green (Takara) protocol. α-SMA was used as an internal control. Primers are reported in Table 1.

**Table 1.** Primers of Kv channels.

Gene	Forward	Reverse
Kv1.2	GCTGCCTATTTGTGTATCTGTG	CATTAGTCTGCGCTCCTGTAG
Kv1.5	GACAGTATCAGAAGGGGTAGC	TTTTTACAAATCTGTTTCAC
Kv2.1	GAAACTCTGGGATCTGCTGG	GAACTCGTCTAGGCTCTGC
Kv3.1	CATCTGGGCACTGTTCGAG	TCTGTCTTGTTCACGATGGG
Kv9.3	TGAGCCACTAAATGCCGATG	GAAGATCACGCTGAACCCTC

### Immunoprecipitation and immunoblotting

The smooth muscle layer of resistance PA rings was dissected. Total protein extracts and protein concentrations were prepared as previously described (2).

Immunoprecipitations were performed as follows: Lysates containing 0.5 mg total protein were incubated with 10 μg Kv1.2 (APC-010; Alomone Lab, Israel), Kv1.5 (APC-004), Kv2.1 (APC-012), Kv3.1b (APC-014), or Kv9.3 (HPA014864; Sigma, USA) monoclonal antibody overnight at 4°C, which were then captured by Protein A-Sepharose CL-4B beads (Santa Cruz Biotechnology, USA). The beads were collected by centrifugation at 200 *g* for 3 min at 4°C followed by 3 washes with lysis buffer. After the final wash, the immunoprecipitates were subjected to western blot analyses.

Immunoblotting was performed to detect phosphotyrosine levels in hypoxia-related Kv channels as described previously (24). Membranes were immunoblotted using antibodies to p-Tyr (SC-7020, Santa Cruz Biotechnology) at 4°C overnight. The signal intensity of the immunoreactive p-Tyr bands from the membrane protein was normalized to Kv channel expression. Fluorescent secondary antibodies (Rockland, USA) were visualized using an Odyssey scanner (LI-COR, USA).

### Immunofluorescence

PASMCs were dispersed from 12-week-old rat resistance PAs. After enzymatic isolation, PASMCs were cultured at 4°C in Dulbecco's Modified Eagle's Medium (Corning, USA) supplemented with 15% fetal bovine serum for 2 h to allow cells to adhere to the plates.

Confocal microscopy imaging was performed with an Olympus confocal laser scanning microscope (BX61W1-FV1000, Japan). The Kv1.5 primary antibody (1:100; APC-150; Alomone Lab) and p-Tyr (1:100, SC-7020, Santa Cruz Biotechnology) were used as described previously. The secondary antibodies tetramethylrhodamine isothiocyanate (1:200; ZF-0316; ZSGB) and fluorescein isothiocyanate (1:150; A0568; Beyotime, China) were used. Nuclear staining was performed using 4′,6′-diamidino-2-phenylindole dihydrochloride (DAPI; 100 nM; Sigma) in fixed cells as previously described. Images of cells were analyzed using Image Pro Plus software version 5.0 (Media Cybernetics, USA).

### Statistical analysis

Data are reported as mean±SE and analyzed using one-way ANOVA. A P value of <0.05 was considered to be statistically significant.

## Results

### Exaggerated pulmonary hypertension in IUGR-hypoxia rats

**Figure 1.**
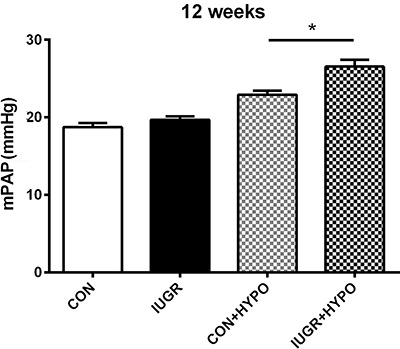
Intrauterine growth retardation (IUGR) and control-hypoxia and normoxia rat mean pulmonary artery pressure (mPAP). CON: control group; IUGR: IUGR group; CON+HYPO: control group with 2-week hypoxia; IUGR+HYPO: IUGR group with 2-week hypoxia. Data are reported as means±SD. *P<0.05 (ANOVA).

The birth weights of the IUGR offspring were significantly lower than that of the control offspring (Control *vs* IUGR: 7.2±0.07 *vs* 5.0±0.10 g, P<0.05). There was no difference in litter size between the two groups (10±2/L), and no significant differences in body weight were observed between the control and IUGR groups at 12 weeks of age (Control *vs* IUGR: 503±10.62 *vs* 483±9.58 g, P=0.19). The mPAP of 12-week-old rats was determined in order to examine the effect of IUGR on hemodynamics. At 12 weeks, the mPAP of the control and the IUGR rats was 18.7±0.53 and 19.7±0.43 mmHg, respectively; no differences were found between the two groups (P=0.18; Figure 1). After 2 weeks of hypoxia, the mPAP of IUGR-hypoxia rats increased to 26.6±2.49 mmHg, while that of control-hypoxia rats increased to 22.9±1.52 mmHg (P<0.05, Figure 1).

**Figure 2.**
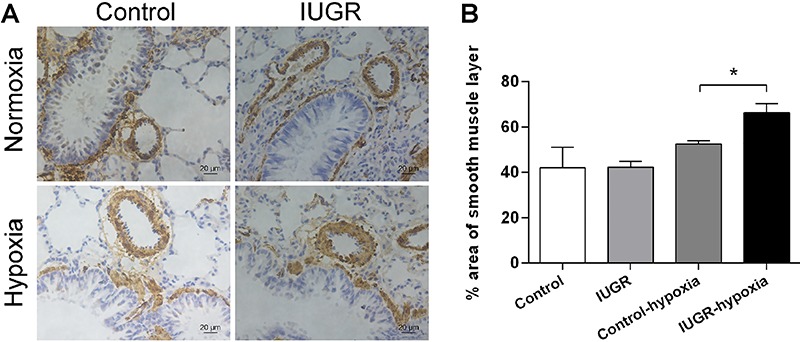
Remodeling of resistance pulmonary artery (PA) ring smooth muscle layers of intrauterine growth retardation (IUGR)-hypoxia and control-hypoxia rats. *A*, Representative immunohistochemical staining images of smooth muscle layers of resistance PAs. Brown indicates α-SMA (smooth muscle actin). Scale bar=20 µm. *B*, Summary histogram of immunohistochemical staining images of the smooth muscle layer of all groups. The smooth muscle layer of resistance PA rings is thicker in IUGR rats than in control rats after 2 weeks of hypoxia. Data are reported as means±SD. *P<0.05 (ANOVA).

Immunohistochemical staining of lung tissue was performed. There was no difference in the percent thickness of the smooth muscle layer of PA rings in control rats (42.1±9.0%) and IUGR rats (42.2±2.7%). After 2 weeks of hypoxia, the smooth muscle layer became thicker in both groups, but that of the IUGR-hypoxia group rats became significantly thicker than that of the control-hypoxia group (66.3±3.9 and 52.6±1.4%, respectively; Figure 2).

### Kv1.5 expression in IUGR pulmonary artery smooth muscle decreased after hypoxia

**Figure 3.**
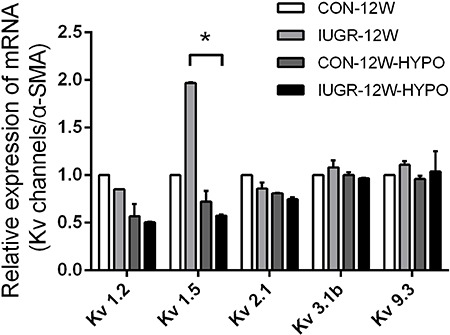
Pulmonary artery smooth muscle cells (PASMCs) Kv channel mRNA levels in hypoxia and normoxia rats. CON-12W: control rats at 12 weeks old; IUGR-12W: intrauterine growth retardation (IUGR) rats at 12 weeks old; CON-12W-HYPO: control rats at 12 weeks old with 2 weeks of hypoxia; IUGR-12W-HYPO: IUGR rats at 12 weeks old with 2 weeks of hypoxia. Data are reported as means±SD. *P<0.05 (ANOVA).

The mRNA expression of Kv channels (Kv1.2, Kv1.5, Kv2.1, Kv3.1b, Kv9.3) in pulmonary artery smooth muscle was examined in both the normoxia and hypoxia groups. There was no significant difference in mRNA expression of Kv1.2, Kv2.1, Kv3.1b and Kv9.3 between control rats and IUGR rats, while that of Kv1.5 increased nearly 2-fold in IUGR rats. A significant decrease of Kv1.5 mRNA level was seen between IUGR-normoxia and IUGR-hypoxia groups (P<0.05). However, no difference was detected in mRNA expression of Kv1.5 between control-hypoxia and IUGR-hypoxia groups (Figure 3).

### Increased tyrosine phosphorylation of Kv1.5 in PASMCs of IUGR rats after hypoxia

**Figure 4.**
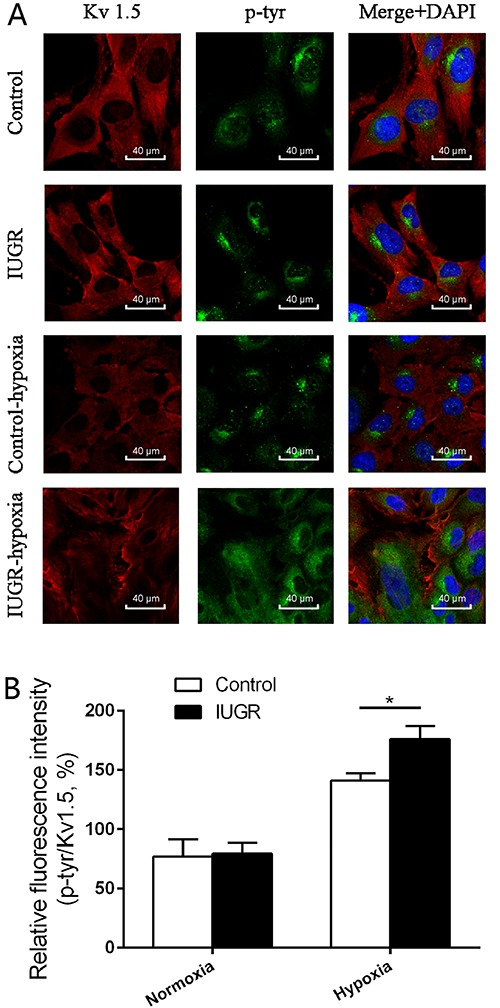
Immunofluorescence detection of Kv1.5 and tyrosine-phosphorylated Kv1.5 in pulmonary artery smooth muscle cells PASMCs. *A*, Kv1.5 expression (red) is downregulated and tyrosine-phosphorylated Kv1.5 expression (green) is upregulated to varying degrees in both intrauterine growth retardation (IUGR) rats and control rats after 2 weeks of hypoxia. Nuclei are stained with DAPI. Scale bar=20 µm. *B*, Summary histogram of relative fluorescence intensity of p-tyr/Kv1.5. Data are reported as means±SD. *P<0.05 (ANOVA).

In order to investigate tyrosine phosphorylation of Kv channels in PASMCs, fluorescence detection and immunoblotting were performed. The fluorescence intensity of Kv1.5 tyrosine phosphate in separated PASMCs increased after 2 weeks of hypoxia in both the control and IUGR rats, but expression was much higher in the IUGR-hypoxia group than in the control-hypoxia group (Figure 4).

**Figure 5.**
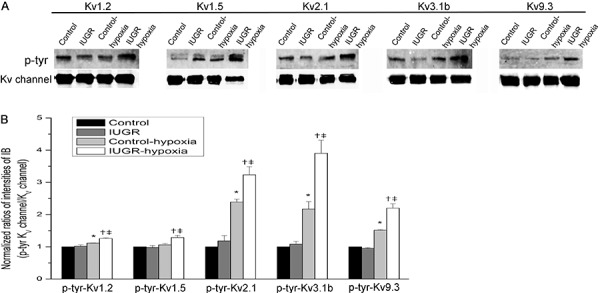
Co-immunoprecipitation analysis of the effect of hypoxia on tyrosine phosphorylation of Kv channels in pulmonary artery smooth muscle cells. *A*, Representative immunoblots of the α-subunit of Kv channels and their relevant phosphotyrosine (p-tyr). *B*, Summary histogram of immunoblot (IB) analysis of Kv channel p-tyr. Data are reported as means±SD. *P<0.05, control groups *vs* control-hypoxia groups; ^†^P<0.05 intrauterine growth retardation (IUGR) groups *vs* IUGR-hypoxia groups; ^‡^P<0.05, control-hypoxia groups *vs* IUGR-hypoxia groups (ANOVA).

We then performed co-immunoprecipitation with antibodies specific to tyrosine-phosphorylated Kv1.2, Kv1.5, Kv2.1, Kv3.1b, and Kv9.3. Expression of tyrosine-phosphorylated Kv channels was upregulated after hypoxia, and the differences were more significant in the IUGR-hypoxia group than in the control-hypoxia group (Figure 5).

## Discussion

PAH in adulthood is associated with intrauterine malnutrition (1,2). Our previous research suggests that the potassium channel Kv1.5 of PASMCs plays an important role in this phenomenon.

Many studies have demonstrated the PAH novel concept of “fetal origin of adult disease”. However, the activities of *I*_K_ are dependent not only on expression levels of the potassium channel, but also on the phosphorylation status of the channels (25). Therefore, quantification of differences in the phosphorylation status of this pivotal potassium channel, Kv1.5, can reflect changes in *I*_K_, which in turn indicates the degree of PASMCs proliferation in rats following hypoxia. Hence, regulating the amplitude of *I*_K_ may be a feasible strategy to minimize PASMCs proliferation following hypoxia in adulthood due to IUGR.

Here, we found that IUGR rats developed exaggerated pulmonary hypertension and a thickened smooth muscle layer as adults, while expression of the Kv1.5 channel significantly decreased. The mRNA expression of Kv1.5 decreased significantly in IUGR rats after 2 weeks of hypoxia, while all the other Kv channels showed no differences. Immunoblotting analysis revealed that the tyrosine phosphorylation level of all Kv channels included in this study increased more significantly after hypoxia in IUGR-hypoxia rats than in control-hypoxia rats. In our previous study, however, we only found that expression of the Kv1.5 α-protein was significantly downregulated (9). These data demonstrate that tyrosine phosphorylation plays an important role in Kv1.5 expression in the PASMCs of IUGR rats with exaggerated PAH in adulthood. The variation tendency of Kv1.5 α-protein and mRNA was coincident, while protein expression levels were much lower. This provides new insight into post-transcriptional regulation in the IUGR-hypoxia pathophysiological process. As previous studies have reported, tyrosine phosphorylation of many potassium channels such as Kv1.2 and Kv1.5 could suppress channel activity by using protein tyrosine kinases to bind and phosphorylate the potassium channels (21–23). Here, we revealed a mechanism underlying the downregulation of Kv1.5 expression. Though the tyrosine phosphorylation level of all Kv channels increased in IUGR-hypoxia rats, only Kv1.5 expression significantly decreased in IUGR-hypoxia rats. Therefore, although the amplitude and inactivation kinetics of *I*_K_ has not been detected, we speculate that the tyrosine phosphorylation functions as an important mechanism in Kv1.5 α-protein over-expression, which causes an increase in PASMCs proliferation. Although expression of Kv1.2, Kv2.1, Kv3.1, and Kv9.3 p-Tyr increased in IUGR-hypoxia groups compared to control-hypoxia groups, only Kv1.5 α-protein levels decreased more significantly than the mRNA decreased, indicating that the Kv1.5 channel is more sensitive than other Kv channels to the role of tyrosine phosphorylation.

Presently, it is unknown whether tyrosine phosphorylation of Kv1.5 α-protein alone regulates Kv1.5 expression. It is possible that channel activity will also be suppressed by tyrosine phosphorylation, as is the case with tyrosine phosphorylation of Kv1.5 reducing *I*_K_ in astrocytes. Furthermore, tyrosine phosphorylation of Kv1.5 may serve as an adaptor molecule by linking other regulatory components to Kv1.5 in IUGR-hypoxia pathophysiological processes, such as the epigenetic mechanisms that we have reported (2).

Taken together, our results demonstrate that Kv1.5 served as an *in vivo* target in IUGR rats that are subjected to hypoxia in adulthood. In addition, modulation of Kv1.5 channel expression may rely on the tyrosine phosphorylation of Kv1.5 α-protein.
